# ET-26 hydrochloride (ET-26 HCl) has similar hemodynamic stability to that of etomidate in normal and uncontrolled hemorrhagic shock (UHS) rats

**DOI:** 10.1371/journal.pone.0183439

**Published:** 2017-08-15

**Authors:** Bin Wang, Shouming Chen, Jun Yang, Linghui Yang, Jin Liu, Wensheng Zhang

**Affiliations:** 1 Laboratory of Anesthesia and Critical Care Medicine, Translational Neuroscience Center, West China Hospital, Sichuan University, Chengdu, Sichuan, P.R. China; 2 Department of Anesthesiology, West China Hospital, Sichuan University, Chengdu, Sichuan, P.R. China; 3 Department of Anesthesiology, Second Affiliated Hospital, Zhejiang University School of Medicine, Hangzhou, P. R. China; McLean Hospital/ Harvard Medical School, UNITED STATES

## Abstract

**Objective:**

ET-26 HCl is a promising sedative–hypnotic anesthetic with virtually no effect on adrenocortical steroid synthesis. However, whether or not ET-26 HCl also has a sufficiently wide safety margin and hemodynamic stability similar to that of etomidate and related compounds remains unknown. In this study, the effects of ET-26 HCl, etomidate and propofol on therapeutic index, heart rate (HR), mean arterial pressure (MAP), maximal rate for left ventricular pressure rise (D_max_/t), and maximal rate for left ventricular pressure decline (D_min_/t) were investigated in healthy rats and a rat model of uncontrolled hemorrhagic shock (UHS).

**Methods:**

50% effective dose (ED_50_) and 50% lethal dose (LD_50_) were determined after single bolus doses of propofol, etomidate, or ET-26 HCl using the Bliss method and the up and down method, respectively. All rats were divided into either the normal group and received either etomidate, ET-26 HCl or propofol, (*n* = 6 per group) or the UHS group and received either etomidate, ET-26 HCl or propofol, (*n* = 6 per group). In the normal group, after preparation for hemodynamic and heart-function monitoring, rats were administered a dose of one of the test agents twofold-higher than the established ED_50_, followed by hemodynamic and heart-function monitoring. Rats in the UHS group underwent experimentally induced UHS with a target arterial pressure of 40 mmHg for 1 hour, followed by administration of an ED_50_ dose of one of the experimental agents. Blood-gas analysis was conducted on samples obtained during equilibration with the experimental setup and at the end of the experiment.

**Results:**

In the normal group, no significant differences in HR, MAP, D_max_/t and D_min_/t (all *P* > 0.05) were observed at any time point between the etomidate and ET-26 HCl groups, whereas HR, MAP and D_max_/t decreased briefly and D_min_/t increased following propofol administration. In the UHS group, no significant differences in HR, MAP, D_max_/t and D_min_/t were observed before and after administration of etomidate or ET-26 HCl at ED_50_ doses (all *P* > 0.05). Administration of propofol resulted in brief, statistically significant reductions in HR and D_max_/t, with a brief increase in D_min_/t (*P* ˂ 0.05), while no significant differences in MAP were observed among the three groups. The blood-lactate concentrations of rats in the ET-26 HCl group were significantly lower than those in etomidate and propofol groups (*P* ˂ 0.05).

**Conclusions:**

ET-26 HCl provides a similar level of hemodynamic stability to that obtained with etomidate in both healthy rats, and rat models of UHS. ET-26 HCl has the potential to be a novel induction anesthetic for use in critically ill patients.

## 1. Introduction

An abundance of evidence indicates that hypotension has an adverse effect on critically ill patients during the perioperative period. Anesthesia-induced hemodynamic collapse, owing to hypotension, is life-threatening in such patients [1,2], who have a higher mortality rate than that of patients whose blood pressure remains stable during anesthesia [3]. Exposure to trauma leads to hemorrhagic shock, which requires surgical intervention to control the bleeding. Induction of anesthesia using the commonly used anesthetic propofol carries an increased risk of hypotension, resulting in cardiovascular adverse effects [4]. Etomidate can be used as an alternative to propofol in order to prevent, or reduce the risk of hemodynamic instability during the induction of anesthesia. The incidence of hypotension is substantially lower among patients in critical care who are anesthetized using etomidate compared with those whose anesthesia is induced using propofol or midazolam. Furthermore, etomidate-induced hypotension is less severe, and easier to ameliorate than that induced by propofol or midazolam [5–7]. Although etomidate has virtually no effect on hemodynamic stability [8,9], the suppression of corticosteroid synthesis associated with this agent limits its use; however, whether or not etomidate-induced suppression of adrenal gland function affects mortality remains controversial [10–14]. Therefore, the development of a novel anesthetic that has the hemodynamic stability of etomidate without affecting corticosteroid synthesis is an important step in the development of safer anesthetics.

ET-26 hydrochloride (ET-26 HCl) is an etomidate analogue with similar hypnotic properties to those of etomidate, which has been previously shown to have virtually no effect on adrenocortical steroid synthesis [15,16]. These observations suggest that ET-26 HCl is a safer anesthetic than propofol or etomidate, and could be used to anesthetize patients while preserving hemodynamic stability, even in patients undergoing hemorrhagic shock.

In this study, we investigated the ED_50_ and LD_50_ of ET-26 HCl, etomidate and propofol in tracheally intubated rats. We also investigated the effects of ET-26 HCl, propofol and etomidate on hemodynamic stability in rats with blood pressures in the normal physiological range, and in a rat model of uncontrolled hemorrhagic shock (UHS).

## 2. Methods

The present study adheres to the applicable EQUATOR guidelines on the reporting of health research. All experiments were undertaken with the approval of the Committee of Scientific Research and the Institutional Animal Experimental Ethics Committee of the West China Hospital (Sichuan University, Chengdu, China) and all ethical approval decisions were based on the recommendations in their guidelines (publication number 2015015A).

Adult Sprague–Dawley rats were purchased from Chengdu Dassy Biological Technology Co. Ltd. (Chengdu, China). All animals were housed in the Animal Experimental Center of Sichuan University at an ambient temperature of 25 ± 1°C, with controlled humidity levels of 60% and a 12-h light–dark cycle (from 7 am to 7 pm). All rats had access to water and food *ad libitum*. Rats were acclimatized for 1 week before experimentation.

Etomidate (2 mg/ml) and propofol (10 mg/ml) were purchased from Nhwa Pharma. Corporation (Xuzhou, China) and Astra Zeneca (Caponago Milano, Italy), respectively. ET-26 HCl (10 mg/ml) was synthesized in our laboratory as described in previous article[15].

### 2.1. Determination of ED_50_ for loss of righting reflex (LORR) and LD_50_

The Bliss method was used to measure the ED_50_ of each agent. A total of 50 rats received each drug in order to determine ED_50_. Doses of ET-26 of 1.33, 1.64, 2, 2.48, and 3 mg/kg were used to generate data for measurements. For etomidate, the doses used were 0.48, 0.58, 0.69, 0.83, and 1.0 mg/kg. The doses of propofol used were 4.1, 4.77, 5.55, 6.45, and 7.5 mg/kg of body weight. After injection, rats were monitored for signs of LORR. A period >30 s was considered to indicate the induction of anesthesia.

The up and down method was used to determine LD_50_[17]. Rats received a range of doses of ET-26 HCl (33.8 mg/kg, 27 mg/kg, 21.6 mg/kg or 17.3 mg/kg), propofol (31.6 mg/kg, 25.3 mg/kg, 20.2 mg/kg, or 16.2 mg/kg.) and etomidate (16 mg/kg, 12.8 mg/kg, 10.2 mg/kg, or 8.2 mg/kg). When the animals stopped breathing, electrocardiogram (ECG) waveforms were attached and observed. The complete disappearance of the ECG waveform was regarded as death [18].

### 2.2. Surgery

All rats were anesthetized using a pentobarbital sodium solution (40 mg/kg, intraperitoneally) and mechanically ventilated with a fraction of inspired oxygen of 21%, which was controlled using a rodent ventilator (Chengdu Techman Software Co. Ltd, Chengdu, China). The respiratory parameters were set at 80 breaths per minute, with an inspiration to expiration ratio of 5:4, and a tidal volume of 5 mL. Each rat was placed on a warming pad. The body temperature of each rat was monitored using a rectal probe. For measurements of hemodynamic and heart-function parameters, the femoral artery was isolated and cannulated using a polyethylene catheter fitted with an angiographic needle (18G, BD, Melsungen, German), following a sterile downward incision in the right groin and right neck region. To enable blood pressure monitoring, a catheter was inserted into the common carotid artery and inserted as far as the left ventricle of the heart. A tail-vein catheter (18 G) was inserted to enable drug administration. A BL-420F data acquisition and analysis system was connected to the pressure channel and ECG channel in order to collect data on blood pressure and ECG parameters. After preparation, each rat was allowed 30 minutes to become used to the presence of the measuring devices, and data on MAP, HR, Dmax/t, and Dmin/t were collected every 5 minutes as baseline readings.

### 2.3. Rat models of UHS

After a 30-minute equilibration period, UHS was created using a modified method originally developed by Peter Safer [19]. An initial volume-controlled hemorrhage, resulting in blood loss of 2.5 mL/100g was induced surgically over a 15-minute period, followed by a 15-minute equilibration period. UHS was followed by tail amputation over a 60-minute period. Data on the previously described physiological parameters were collected every 5 minutes during the UHS procedure. The UHS model was considered to be established when MAP was below 40 mmHg.

### 2.4. Drug administration and monitoring

After equilibration for 30 minutes, or establishment of experimentally induced UHS, a single bolus dose of one of the test drugs (either etomidate, propofol, or ET-26 HCl) was given intravenously over a 15-second period. Data on physiological parameters were recorded once every 15 seconds over the 2-minute period immediately after the injection, followed by once every minute until 5 minutes after administration of all drugs. Following this, data were collected at 5-minute intervals.

### 2.5. Blood-gas analysis of UHS rats

The arterial blood samples were collected after establishment of UHS model and at the end of observe period of experiment. The partial pressure of oxygen (pO_2_), carbon dioxide (pCO_2_), the oxygen saturation level of hemoglobin (sO2), concentration of hemoglobin, potassium ion(K^+^), sodium ion (Na^+^), calcium ion (Ca^2+^) and lactic acid (LAC) were analyzed using blood gas analyzer (Mindray, Shenzhen, China).

### 2.6. Data and statistical analyses

Based on preliminary experiments, sample sizes were set at a minimum of six rats in present study. All data presented are the mean ± standard deviation of the mean (SD) unless indicated otherwise. Data for body weight and loss of blood volume were compared using one-way analysis of variance (ANOVA) followed by Tukey’s test. Data on HR, MAP, D_max_/t and D_min_/t were analyzed using repeated-measures two-way ANOVA tests. The statistical significance of pairwise comparisons was investigated using Tukey’s post-hoc test. Differences yielding a *P*-value of ˂ 0.05 were considered statistically significant.

All analyses were performed using SPSS v21.0 statistical analysis software (IBM, Armonk, NY, USA). All figures and dose-response curves were generated using Prism v5.0 analysis software (GraphPad, San Diego, CA, USA) and Photoshop CS5 software (Adobe, San Jose, CA, USA).

## 3. Results

### 3.1 Determination of ED_50_ for LORR and LD_50_

**Fig 1:**
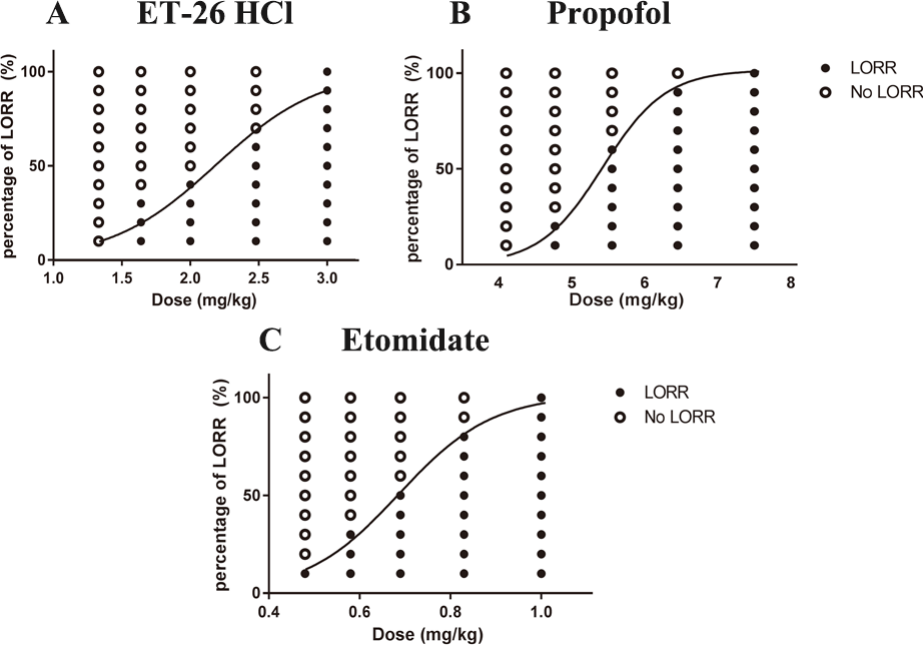
50% median effective dose (ED_50_) for Loss of righting reflex (LORR) of A: ET-26 HCl; B: Propofol; C: Etomidate. Each symbol represents data from one rat. The curve is a fit of the data set using non-linear regression.

The potency of ET-26 HCl (ED_50_ = 2.1 mg/kg), defined by the dose required to induce LORR in 50% of rats, was lower than that of propofol (ED_50_ = 5.4 mg/kg), but greater than that of etomidate (ED_50_ = 0.66 mg/kg). Dose-response curves depicting the effects of these agents are presented in Fig 1. Furthermore, the LD_50_ value of ET-26 (24.1 mg/kg) was greater than that of propofol (22.6 mg/kg) and etomidate (11.9 mg/kg). Therapeutic index values were 11 for ET-26, 4.2 for propofol and 18 for etomidate (Table 1).

**Table 1:** Indexes calculated during the ED_50_ and LD_50_ studies for ET-26 HCl, propofol, and etomidate in the rats.

	**ET-26HCl**	**Propofol**	**Etomidate**
**ED_50_ (mg/kg)**	2.1	5.4	0.66
**With 95% CI**	1.9–2.4	5.0–5.8	0.59–0.74
**LD_50_ (mg/kg)**	24.1	22.6	11.9
**With 95% CI**	23.4–24.9	20.9–25.9	11.5–12.3
**TI**	11	4.2	18

### 3.2 HR, MAP, D_max_/t and D_min_/t of rats under normal conditions

**Fig 2:**
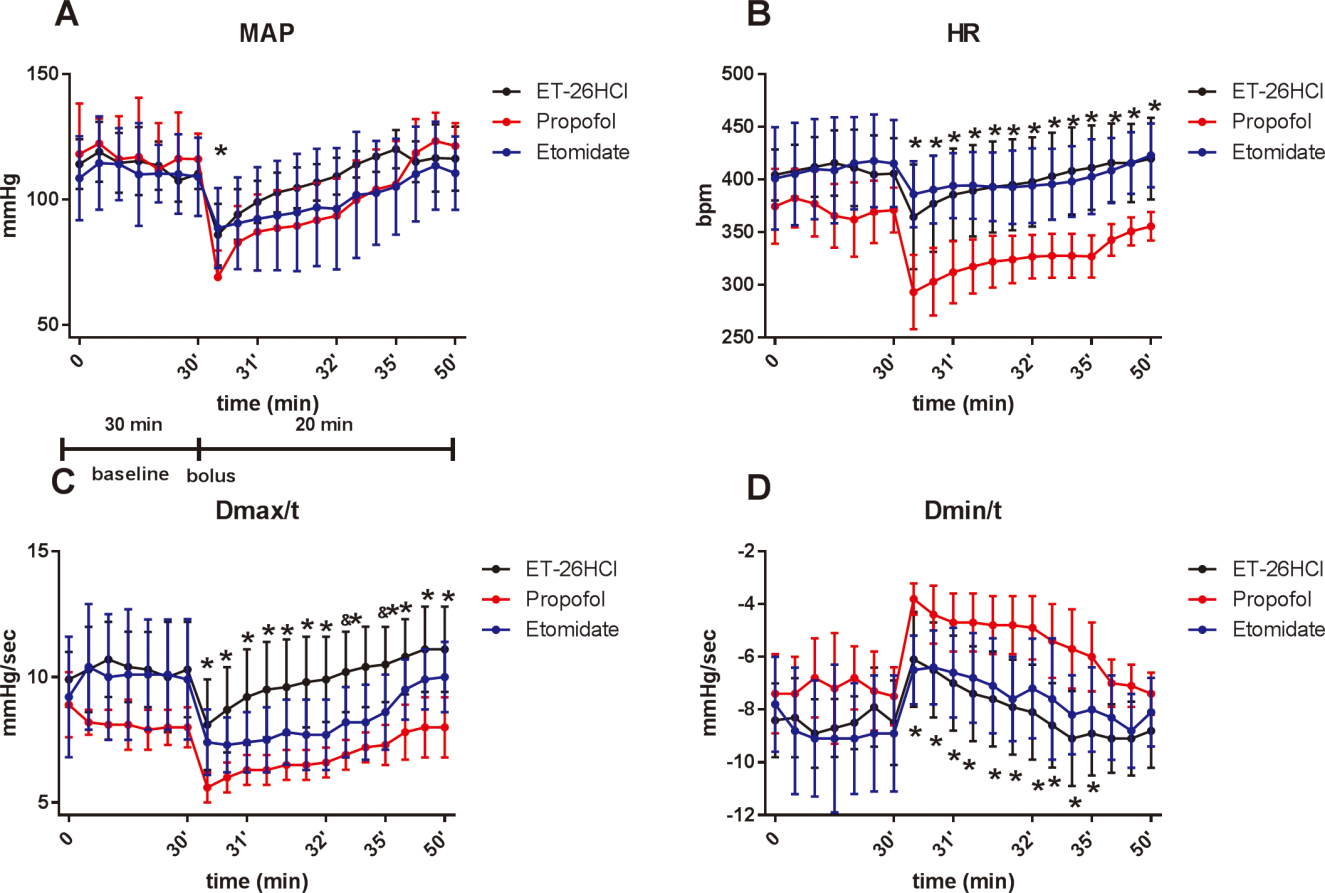
Each data were represented as mean ± SD (*n* = 6 for each group). Results of hemodynamic parameters (MAP: mean arterial pressure; HR: heart rate; Dmax/t; Dmin/t) on Non-UHS rats after dosed with two folds ED_50_ of ET-26 HCl, propofol and etomidate. * P ˂ 0.05 versus propofol group.

Administration of either etomidate, ET-26 HCl or propofol resulted in reductions in HR, MAP and D_max_/t, with a notable increase in D_min_/t (Fig 2). MAP decreased by a mean maximum of 47.0 ± 9.3 mmHg in the propofol group, by 20.3 ± 7.7 mmHg in the etomidate group and by 24.3 ± 9.6 mmHg in the ET-26 HCl group. No significant differences in MAP were observed between rats in the etomidate and ET-26 HCl groups at any point in the experiment (all *P* ˃ 0.05).

Administration of the three drugs resulted in a notable decrease in HR at 15 seconds after injection. The maximum mean decreases in HR was 77.6 ± 23.0 beats per minute (bpm) in the propofol group, 29.1 ± 11.5 bpm in the etomidate group and 41.0 ± 29.3 bpm in the ET-26 HCl group. No significant differences in HR were observed among rats receiving etomidate or those receiving ET-26 HCl; however, the HR of rats in the propofol group was kept lower than that of rats in the other two groups from the first timepoint to the final timepoint (*P* < 0.01).

D_max_/t decreased in the propofol group immediately after injection and did not recover to baseline levels until 3 minutes after injection. D_max_/t values in the etomidate group differed significantly from those of the ET-26 HCl group during the entire observation period, with the exception of the third minute after injection (*P* = 0.037), whereas D_max_/t in the propofol group was lower than that of the etomidate and ET-26 HCl groups.

D_min_/t increased immediately after injection of propofol and recovered at 4 minutes after injection. D_min_/t in the propofol group was significantly higher than that of the ET-26 HCl group at 5 minutes after administration, but not at other timepoints.

### 3.3 HR, MAP, D_max_/t and D_min_/t of rats in UHS rats

**Fig 3:**
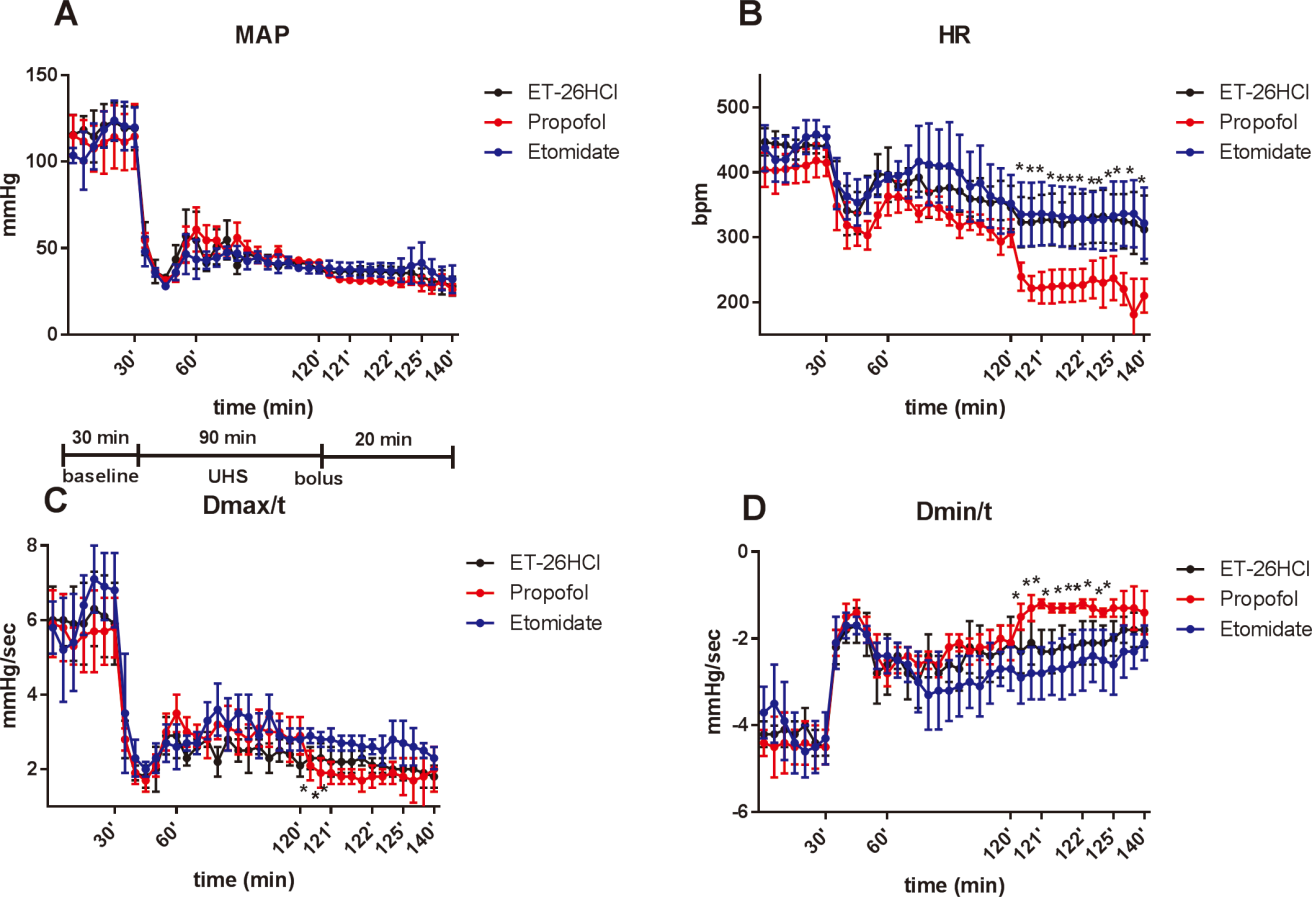
Each data were represented as mean ± SD. Changes of hemodynamic parameters on UHS rats after injection with ED_50_ of ET-26 HCl (*n* = 6), propofol (*n* = 6 for each time point except: *n* = 4 at 15 min and *n* = 3 at 20 min after administration of propofol) and etomidate (*n* = 6). * P ˂ 0.05 versus propofol group.

No significant differences were observed in body weight and volume of blood loss among UHS rats receiving either of the three test drugs (Table 2, Fig 3). During both the operative period and hemorrhagic phase, no notable differences were observed between rats scheduled to receive either etomidate, ET-26 HCl or propofol. However, after injection with propofol at an ED_50_ dose, three of the six rats died under assisted ventilation (at 8, 15, and 19 minutes after administration of propofol) while rats in the ET-26 HCl and etomidate groups all survived.

**Table 2:** The body weight and loss volume of blood (*n* = 6 for each group).

	Etomidate	ET-26 HCl	Propofol	P value
Weight(g)	292.1±20.4	272.3±16.9	267.5±23.7	NS
Loss volume(mL)	7.7±0.8	7.4±0.4	7.1±0.8	NS
Loss volume(mL/100g)	2.6±0.3	2.6±0.2	2.7±0.1	NS

NS means no significant differences were observed among three groups.

Similar to observations from non-UHS rats, HR and D_max_/t of rats in the propofol group decreased markedly compared with the ET-26 HCl and etomidate groups, with an increase in D_min_/t also observed. Unexpectedly, no statistically significant differences in MAP were observed between the three groups.

### 3.4 Blood-gas analysis of UHS rats

No statistically significant differences were detected on blood-gas analysis among the three groups following establishment of UHS. Blood lactate acid concentrations in the ET-26 HCl group were significantly lower than those of the etomidate or propofol groups (*P* < 0.01) at the end of experiment (Table 3). No significant differences were observed in comparisons of other blood-gas parameters.

**Table 3:** The arterial blood gas analysis results of rats before administration of test drugs (*n* = 6 for each group respectively) and 20 min after injection of ED_50_ of etomidate (*n* = 6), ET-26 HCl (*n* = 6) and propofol (*n* = 3) in a rat model of UHS (uncontrolled hemorrhagic shock).

	baseline	Etomidate	ET-26 HCl	Propofol
PH	7.35±0.1	7.17±0.1^a^	7.22±0.1^a^^,^^d^	7.08±0.1^a^^,^^c^
PCO_2_(mmHg)	34.3±8.5	16.2±9.8^a^	20.6±11.6 ^a^	28.6±12.3 ^a^
PO_2_(mmHg)	91.6±12.2	127.3±17.2 ^a^	117.6±7.9 ^a^	119.4±23.4 ^a^
Hb(g/dL)	14.1±0.9	12.9±0.9 ^a^	12.5±0.9 ^a^	11.9±1.2 ^a^
SO_2_(%)	89.6±3.9	87.7±10.2	83.3±16.5	83.3±12.3
Na^+^(mmol/L)	140.5±1.4	138.8±0.8	141.1±1.1	139.8±1.4
Ca^2+^(mmol/L)	1.2±0.0	1.3±0.1	1.3±0.1	1.3±0.1
Cl^-^(mmol/L)	109.8±1.8	112.3±1.1	115.1±2.5	112.2±1.9
Lac(mmol/L)	2.9±1.8	12.8±1.8 ^a^^,^^c^	9.3±1.82 ^a^^,^^b^^,^^d^	10.8±1.9 ^a^^,^^c^
K^+^(mmol/L)	3.8±0.4	5.6±1.3 ^a^	6.3±1.2 ^a^	6.1±1.1 ^a^

^a^*P* ˂ 0.05 versus baseline

^b^*P* ˂ 0.05 versus etomidate

^c^*P ˂* 0.05 versus ET-26 HCl

^d^*P* ˂ 0.05 versus propofol.

## 4. Discussion

In this study, ET-26 HCl was found to preserve hemodynamic stability, as indicated by a wide range of physiological parameters in both healthy rats and in a rat model of UHS. In addition, lactic acid levels in rats receiving ET-26 HCl were significantly lower than those receiving etomidate or propofol.

The results, following tracheal intubation, indicate that ET-26 HCl, similar to etomidate has a wide safety margin. In healthy, hemodynamically stable rats, ET-26 HCl had virtually no effect on blood pressure or cardiac function. To further evaluate the hemodynamic stability of the test compounds, a rat UHS model, which closely imitates the effects of trauma and major bleeding experienced by patients was selected. A volume of blood loss of 3.5 mL/100 g has previously been reported in rat models of UHS [19]. However, based on the findings of our previous preliminary experiments, this volume of blood loss leads to high levels of mortality. Therefore, according to preliminary experimental data, we modified our method of UHS induction, and reduced the volume of blood loss to 2.5 mL/100g. Under these conditions, a higher rate of successful UHS was achieved.

In this study, we used test doses to determine the ED_50_ for LORR. According to clinical experience, the dose used for induction of anesthesia in patients at risk of hemodynamic instability is usually lower than that administered to hemodynamically stable patients. In addition, some researchers report that propofol has a more potent anesthetic effect in experimental animals with a reduced, or low blood volume [20–22]. In previous experiments, six UHS rats all died soon after receiving a dose of propofol twofold higher than the ED_50_ dose. Therefore, in this study, we selected a dose equivalent to the ED_50_ dose, defined by LORR. However, this observation was made in the presence of a basic level of anesthesia with pentobarbital and we did not monitor the depth of anesthesia, therefore, whether or not the ED_50_ dose would achieve a suitable depth of anesthesia during intubation remains unclear. We suggest that investigations involving telemetry techniques should be applied in order to further define the hemodynamic parameters relating to a response to propofol.

In order to establish uniform baseline parameters, we obtained samples for arterial blood-gas analysis of all rats during the establishment of the UHS model. In a state of low blood volume, the oxygen-carrying capacity of the blood is decreased substantially, with a resultant increase in blood-lactate concentration, which is associated with disease severity, and lower levels of lactic acid are associated with a more favorable prognosis in patients [23,24]. At the end of the experiment, arterial blood-lactate concentrations in the etomidate group were significantly higher than those of the ET-26 HCl group. Therefore, we speculate that ET-26 HCl provides certain advantages over etomidate, such as improved microcirculation and tissue oxygen supply. However, further experiments are required to fully explore the possibility of such effects.

In addition, there are some limitations in this study. We adopted a single blinded investigation instead of a double-blinded one. The propofol group would be easy to identify potentially introducing bias. Besides, there was no control group, so the actual effects of each of the sedatives remain unknown. In summary, ET-26 HCl seems to be a suitable anesthetic for critically ill patients, especially those at risk of hemodynamic instability following induction of anesthesia.

## Supporting information

S1 Table Hemodynamic parameters of non-uncontrolled hemorrhagic shock (UHS) rats after administration of etomidate, ET-26HCl and propofol. (DOCX)

S2 Table Hemodynamic parameters of uncontrolled hemorrhagic shock (UHS) rats after administration of etomidate, ET-26HCl and propofol. (DOCX)
