# Supplementary material for: ET-26 hydrochloride (ET-26 HCl) has similar hemodynamic stability to that of etomidate in normal and uncontrolled hemorrhagic shock (UHS) rats
Source: PLoS One. 2017 Aug 15;12(8):e0183439. doi: 10.1371/journal.pone.0183439 (PMC5557577; doi:10.1371/journal.pone.0183439)
Supplement: S1 Table — (DOCX) [file pone.0183439.s001.docx]

Supplementary Table 1. Hemodynamic parameters of non-uncontrolled hemorrhagic shock (UHS) rats after administration of etomidate, ET-26HCl and propofol.

| time | MAP(mmHg) | | | HR(bpm) | | | D_max_/t（mmHg/ms） | | | D_min_/t（mmHg/ms） | | |
| --- | --- | --- | --- | --- | --- | --- | --- | --- | --- | --- | --- | --- |
|  | ET | ET-26 HCl | PROP | ET | ET-26 HCl | PROP | ET | ET-26 HCl | PROP | ET | ET-26 HCl | PROP |
| 0 | 108.5±16.7 | 114.1±9.9 | 118.1±20.1 | 401.0±48.6 | 404.2±24.3 | 374.5±35.5 | 9.2±2.4 | 9.9±1.1 | 8.9±1.3 | -7.8±1.8 | -8.4±1.4 | -7.4±1.5 |
| 5’ | 114.5±18.6 | 119.0±11.9 | 122.3±9.9 | 405.0±48.6 | 408.3±24.7 | 382.1±27.8 | 10.4±2.5 | 10.3±1.7 | 8.2±0.5 | -8.8±2.4 | -8.3±1.5 | -7.4±1.4 |
| 10’ | 114.1±14.4 | 114.6±11.9 | 116.1±17.1 | 409.6±47.6 | 411.8±28.3 | 377.0±31.0 | 10.0±2.5 | 10.7±1.5 | 8.1±0.6 | -9.1±2.2 | -8.9±1.3 | -6.8±1.5 |
| 15’ | 110.0±20.5 | 115.3±13.6 | 117.0±23.6 | 408.6±50.3 | 415.5±31.1 | 365.5±30.3 | 10.1±2.6 | 10.4±1.4 | 8.1±1.0 | -9.1±2.8 | -8.7±1.1 | -7.2±2.1 |
| 20’ | 110.3±11.5 | 113.5±9.7 | 112.1±18.4 | 415.3±43.7 | 411.0±36.3 | 362.0±35.3 | 10.1±2.2 | 10.3±1.5 | 7.9±0.8 | -9.1±2.1 | -8.5±1.0 | -6.8±1.2 |
| 25’ | 110.1±15.9 | 107.5±8.4 | 116.3±18.4 | 417.5±44.1 | 404.6±37.2 | 369.3±29.6 | 10.1±2.2 | 10.0±2.2 | 8.0±0.7 | -8.9±2.2 | -7.9±1.5 | -7.3±1.5 |
| 30’ | 109.0±15.6 | 110.2±6.1 | 116.1±10.2 | 415.1±41.2 | 405.5±33.6 | 371.0±21.3 | 9.9±2.4 | 10.3±1.9 | 8.0±0.8 | -8.9±2.2 | -8.5±1.6 | -7.5±1.1 |
| 30’30” | 88.6±15.9 | 86.0±12.3 | 69.1±10.6 | 386.0±31.3 | 364.5±49.7 | 293.3±35.3 | 7.4±1.3 | 8.1±1.8 | 5.6±0.6 | -6.5±1.3 | -6.1±1.8 | -3.8±0.6 |
| 30’45’’ | 90.5±18.3 | 94.0±10.1 | 82.8±14.6 | 390.3±32.1 | 377.0±45.7 | 303.1±32.1 | 7.3±1.1 | 8.7±1.7 | 6.0±0.6 | -6.4±1.4 | -6.5±1.8 | -4.4±1.1 |
| 31’ | 92.3±20.6 | 99.0±8.4 | 87.1±14.9 | 394.1±30.8 | 385.5±43.7 | 312.0±29.3 | 7.4±1.2 | 9.2±1.9 | 6.3±0.6 | -6.6±1.7 | -7.0±1.8 | -4.7±1.1 |
| 31’15’’ | 93.6±21.8 | 102.8±7.9 | 88.6±15.1 | 394.3±31.7 | 389.3±43.4 | 317.3±25.7 | 7.5±1.3 | 9.5±1.9 | 6.3±0.6 | -6.8±1.7 | -7.4±1.8 | -4.7±1.1 |
| 31’30’’ | 94.8±23.4 | 104.6±8.2 | 89.5±14.9 | 393.1±32.5 | 392.8±43.2 | 322.0±24.6 | 7.8±1.3 | 9.6±1.9 | 6.5±0.6 | -7.1±1.8 | -7.6±1.8 | -4.8±1.1 |
| 31’45’’ | 96.8±23.4 | 106.8±7.3 | 91.8±14.8 | 392.6±34.8 | 394.8±43.3 | 324.1±22.5 | 7.7±1.4 | 9.8±1.8 | 6.5±0.6 | -7.6±1.6 | -7.9±1.8 | -4.8±1.1 |
| 32’ | 96.3±24.2 | 109.3±7.4 | 93.5±14.8 | 394.1±35.5 | 397.6±42.3 | 326.8±20.7 | 7.7±1.4 | 9.9±1.7 | 6.6±0.6 | -7.2±1.9 | -8.1±1.8 | -4.9±1.2 |
| 33’ | 101.8±25.1 | 114.0±5.3 | 99.8±15.8 | 395.6±33.6 | 403.1±41.3 | 327.5±21.0 | 8.2±1.4 | 10.2±1.6 | 6.9±0.6 | -7.6±2.3 | -8.6±1.6 | -5.4±1.4 |
| 34’ | 102.6±20.7 | 117.1±6.2 | 104.0±15.9 | 398.0±33.6 | 408.1±41.3 | 327.6±20.8 | 8.2±1.5 | 10.4±1.6 | 7.2±0.6 | -8.2±1.5 | -9.1±1.8 | -5.7±1.5 |
| 35’ | 105.1±19.1 | 120.0±7.6 | 106.0±17.3 | 402.5±35.5 | 411.1±40.1 | 327.0±20.1 | 8.6±1.5 | 10.5±1.5 | 7.3±0.8 | -8.0±1.6 | -8.9±1.6 | -6.0±1.3 |
| 40’ | 110.1±18.9 | 115.0±8.2 | 118.5±13.5 | 408.5±30.8 | 415.8±37.3 | 342.5±15.1 | 9.5±1.2 | 10.8±1.5 | 7.8±1.1 | -8.3±1.6 | -9.1±1.3 | -7.0±0.9 |
| 45’ | 113.5±17.5 | 116.5±13.4 | 123.3±11.3 | 415.5±29.1 | 415.5±37.2 | 350.8±13.5 | 9.9±1.2 | 11.1±1.7 | 8.0±1.2 | -8.8±1.4 | -9.1±1.4 | -7.1±0.8 |
| 50’ | 110.5±14.6 | 116.3±12.8 | 121.3±9.1 | 422.8±30.2 | 419.6±38.8 | 355.6±13.6 | 10.0±1.4 | 11.1±1.7 | 8.0±1.2 | -8.1±1.3 | -8.8±1.4 | -7.4±0.8 |
