# Supplementary material for: ET-26 hydrochloride (ET-26 HCl) has similar hemodynamic stability to that of etomidate in normal and uncontrolled hemorrhagic shock (UHS) rats
Source: PLoS One. 2017 Aug 15;12(8):e0183439. doi: 10.1371/journal.pone.0183439 (PMC5557577; doi:10.1371/journal.pone.0183439)
Supplement: S2 Table — (DOCX) [file pone.0183439.s002.docx]

Supplementary Table 2. Hemodynamic parameters of uncontrolled hemorrhagic shock (UHS) rats after administration of etomidate, ET-26HCl and propofol.

| time | MAP(mmHg) | | | HR(bpm) | | | D_max_/t（mmHg/ms） | | | D_min_/t（mmHg/ms） | | |
| --- | --- | --- | --- | --- | --- | --- | --- | --- | --- | --- | --- | --- |
|  | ET | ET-26 HCl | PROP | ET | ET-26 HCl | PROP | ET | ET-26 HCl | PROP | ET | ET-26 HCl | PROP |
| 0 | 103.6±4.4 | 115.6±11.6 | 115.1±11.8 | 437.5±35.5 | 447.0±21.0 | 404.2±26.3 | 5.8±0.7 | 6.0±0.9 | 5.9±0.9 | -3.7±0.6 | -4.2±0.3 | -4.4±0.3 |
| 5’ | 100.5±16.8 | 118.3±8.0 | 111.8±12.2 | 419.1±33.3 | 443.8±19.8 | 403.0±36.0 | 5.2±1.4 | 6.0±0.7 | 5.8±0.9 | -3.5±0.9 | -4.2±0.2 | -4. 5±0.7 |
| 10’ | 108.8±13.2 | 114.6±15.0 | 107.5±13.3 | 420.0±35.6 | 442.5±15.0 | 405.0±23.6 | 5.4±1.3 | 5.9±1.0 | 5.3±0.5 | -3.9±0.9 | -4.1±0.4 | -4.4±0.7 |
| 15’ | 118.5±10.6 | 121.0±12.3 | 111.1±18.1 | 439.5±29.3 | 435.1±15.5 | 409.3±25.8 | 6.4±0.8 | 5.9±1.1 | 5.6±1.0 | -4.4±0.7 | -4.2±0.3 | -4.5±0.4 |
| 20’ | 123.8±11.5 | 123.1±11.1 | 114.3±18.3 | 454.5±26.1 | 442.3±11.2 | 410.8±25.5 | 7.1±0.9 | 6.3±1.0 | 5.7±1.1 | -4.6±0.6 | -4.0±0.4 | -4.4±0.5 |
| 25’ | 120.5±14.0 | 119.3±12.6 | 111.3±16.3 | 458.8±21.8 | 442.3±12.7 | 418.5±26.0 | 6.9±0.9 | 6.1±1.1 | 5.7±0.9 | -4.5±0.6 | -4.4±0.3 | -4.5±0.5 |
| 30’ | 119.8±11.5 | 119.3±13.6 | 114.5±18.8 | 454.5±16.1 | 439.5±15.6 | 414.8±20.5 | 6.8±1.0 | 5.9±1.1 | 5.8±0.8 | -4.3±0.6 | -4.5±0.2 | -4.5±0.4 |
| 35’ | 48.0±8.6 | 55.5±9.5 | 54.6±4.3 | 382.8±39.5 | 382.6±35.1 | 348.1±37.1 | 3.5±1.6 | 2.8±0.5 | 2.8±0.3 | -2.1±0.6 | -2.2±0.4 | -2.1±0.3 |
| 40’ | 36.0±2.6 | 36.5±6.8 | 37.1±2.1 | 363.3±35.4 | 341.5±38.1 | 318.5±35.6 | 2.3±0.5 | 1.9±0.2 | 1.9±0.3 | -1.7±0.3 | -1.8±0.3 | -1.5±0.3 |
| 45’ | 28.1±1.8 | 33.0±1.3 | 31.6±1.6 | 353.8±35.8 | 337.5±32.3 | 312.6±25.6 | 2.0±0.2 | 1.8±0.3 | 1.7±0.3 | -1.7±0.2 | -1.7±0.4 | -1.4±0.3 |
| 50’ | 36.0±4.6 | 43.5±8.3 | 35.6±5.1 | 364.6±28.2 | 366.1±28.8 | 303.1±22.2 | 2.3±0.4 | 2.0±0.6 | 2.1±0.3 | -1.9±0.2 | -1.9±0.5 | -1.8±0.3 |
| 55’ | 46.6±11.5 | 57.0±15.3 | 52.0±10.6 | 382.1±19.7 | 397.3±31.3 | 334.0±19.6 | 2.7±0.5 | 2.9±0.5 | 3.0±0.5 | -2.4±0.3 | -2.8±0.7 | -2.4±0.5 |
| 60’ | 43.5±11.3 | 54.6±16.6 | 60.8±12.7 | 392.5±6.8 | 396.6±42.0 | 363.1±29.4 | 2.6±0.6 | 2.9±0.6 | 3.5±0.5 | -2.4±0.4 | -2.6±0.7 | -2.8±0.3 |
| 65’ | 43.0±5.0 | 41.4±4.7 | 54.5±8.8 | 395.3±22.1 | 378.6±20.0 | 362.5±24.3 | 2.7±0.2 | 2.3±0.2 | 3.0±0.4 | -2.5±0.4 | -2.4±0.3 | -2.5±0.3 |
| 70’ | 44.6±5.3 | 50.8±10.1 | 54.5±8.1 | 400.8±40.8 | 384.5±22.0 | 357.8±15.2 | 2.7±0.3 | 2.6±0.2 | 2.9±0.3 | -2.6±0.4 | -2.8±0.6 | -2.4±0.2 |
| 75’ | 47.6±3.4 | 54.8±11.3 | 47.6±3.1 | 416.8±55.1 | 392.6±22.2 | 336.5±12.8 | 3.3±0.5 | 2.8±0.2 | 2.8±0.5 | -3.0±0.7 | -3.0±0.6 | -2.6±0.2 |
| 80’ | 47.0±4.3 | 40.0±5.0 | 56.1±8.8 | 412.5±63.1 | 369.1±39.1 | 351.6±20.0 | 3.6±0.7 | 2.2±0.4 | 3.2±0.4 | -3.3±0.8 | -2.4±0.5 | -2.5±0.2 |
| 85’ | 42.5±4.8 | 47.0±1.6 | 49.1±5.2 | 409.8±56.5 | 374.5±32.1 | 345.3±17.7 | 3.2±0.7 | 2.8±0.3 | 3.1±0.6 | -3.2±0.9 | -2.8±0.4 | -2.6±0.3 |
| 90’ | 44.8±3.4 | 44.5±2.8 | 46.1±4.8 | 410.1±67.5 | 376.8±29.8 | 332.5±14.0 | 3.5±0.5 | 2.5±0.3 | 3.0±0.6 | -3.2±0.7 | -2.6±0.4 | -2.2±0.3 |
| 95’  100’ | 41.5±3.8  41.1±5.4 | 42.1±4.2  39.5±1.8 | 42.6±2.8  48.0±3.2 | 399.8±58.2  378.3±53.8 | 371.0±32.3  359.5±27.3 | 317.3±18.0  325.3±14.0 | 3.4±0.6  2.9±0.6 | 2.5±0.6  2.6±0.2 | 2.8±0.4  3.1±0.5 | -3.1±0.7  -3.0±0.6 | -2.7±0.5  -2.2±0.6 | -2.1±0.2  -2.3±0.3 |
| 105’ | 41.8±3.2 | 41.8±2.9 | 43.1±2.1 | 383.8±57.8 | 358.3±33.3 | 319.8±15.5 | 3.5±0.5 | 2.3±0.4 | 3.0±0.4 | -3.1±0.7 | -2.3±0.6 | -2.2±0.3 |
| 110’ | 38.6±1.3 | 41.6±1.6 | 43.1±2.1 | 363.8±48.8 | 353.3±25.4 | 312.3±16.0 | 2.9±0.4 | 2.5±0.3 | 3.0±0.5 | -2.8±0.7 | -2.4±0.6 | -2.2±0.4 |
| 115’ | 38.6±3.2 | 41.0±1.3 | 41.8±1.1 | 356.0±50.6 | 356.8±30.5 | 293.6±20.6 | 2.8±0.3 | 2.4±0.3 | 2.8±0.6 | -2.7±0.4 | -2.3±0.6 | -2.0±0.3 |
| 120’ | 38.6±3.2 | 37.5±1.6 | 41.8±1.8 | 352.0±44.0 | 346.1±33.3 | 306.6±21.6 | 2.8±0.3 | 2.1±0.3 | 2.9±0.5 | -2.7±0.5 | -2.1±0.6 | -2.1±0.4 |
| 120’30’’ | 38.5±4.3 | 35.6±2.2 | 34.6±1.1 | 335.6±49.4 | 323.5±38.8 | 239.5±21.8 | 2.9±0.2 | 2.3±0.3 | 2.1±0.4 | -2.9±0.6 | -2.3±0.5 | -1.5±0.3 |
| 120’45’’ | 37.6±4.3 | 36.5±1.6 | 32.1±1.1 | 335.5±48.6 | 323.0±37.6 | 221.5±19.1 | 2.8±0.2 | 2.3±0.4 | 1.9±0.4 | -2.8±0.6 | -2.1±0.5 | -1.3±0.3 |
| 121’ | 37.8±3.8 | 36.6±2.0 | 31.5±0.8 | 336.5±48.6 | 326.5±39.1 | 222.5±24.5 | 2.8±0.3 | 2.2±0.4 | 1.9±0.3 | -2.8±0.6 | -2.3±0.5 | -1.2±0.1 |
| 121’15’’ | 37.5±4.5 | 37.1±2.5 | 31.1±1.2 | 334.5±45.3 | 327.5±40.0 | 224.1±26.1 | 2.7±0.2 | 2.2±0.3 | 1.8±0.2 | -2.7±0.6 | -2.3±0.5 | -1.3±0.1 |
| 121’30’’ | 37.6±3.6 | 36.5±2.6 | 31.3±1.4 | 332.0±46.0 | 319.8±34.2 | 225.6±25.3 | 2.7±0.2 | 2.2±0.3 | 1.8±0.2 | -2.7±0.7 | -2.2±0.5 | -1.3±0.1 |
| 121’45’’ | 37.6±4.0 | 36.5±3.3 | 30.8±1.7 | 330.0±47.6 | 326.8±40.5 | 225.5±25.1 | 2.6±0.3 | 2.3±0.2 | 1.7±0.3 | -2.6±0.7 | -2.2±0.4 | -1.3±0.1 |
| 122’ | 37.0±4.3 | 36.0±3.3 | 30.1±1.6 | 328.5±46.1 | 328.5±39.0 | 226.8±24.8 | 2.6±0.2 | 2.1±0.2 | 1.8±0.3 | -2.5±0.7 | -2.1±0.5 | -1.2±0.1 |
| 123’ | 37.3±6.7 | 35.5±3.5 | 30.0±2.6 | 326.0±44.0 | 331.6±40.1 | 235.3±29.0 | 2.5±0.4 | 2.1±0.2 | 1.8±0.2 | -2.4±0.6 | -2.1±0.5 | -1.3±0.2 |
| 124’ | 39.8±10.4 | 35.8±4.1 | 31.0±2.3 | 327.8±48.5 | 332.5±40.6 | 230.0±38.6 | 2.8±0.5 | 2.0±0.2 | 1.9±0.3 | -2.5±0.7 | -2.1±0.4 | -1.4±0.1 |
| 125’ | 41.6±11.8 | 33.5±4.8 | 29.6±4.4 | 333.0±53.3 | 328.6±38.1 | 237.1±34.1 | 2.7±0.6 | 2.0±0.3 | 1.8±0.5 | -2.6±0.7 | -2.0±0.4 | -1.3±0.2 |
| 130’ | 36.3±7.4 | 31.1±5.1 | 27.8±4.1 | 336.5±49.8 | 324.8±42.1 | 220.5±24.6 | 2.6±0.5 | 2.0±0.3 | 1.7±0.6 | -2.3±0.6 | -1.8±0.3 | -1.3±0.4 |
| 135’ | 32.5±6.8 | 30.3±7.0 | 30.7±4.3 | 336.5±51.8 | 322.3±47.8 | 181.2±55.2 | 2.5±0.2 | 1.9±0.4 | 1.8±0.9 | -2.3±0.4 | -1.8±0.4 | -1.3±0.5 |
| 140’ | 32.0±8.0 | 28.0±5.6 | 26.0±3.3 | 321.8±55.1 | 312.3±52.5 | 210.0±26.0 | 2.3±0.3 | 1.8±0.3 | 2.0±0.6 | -2.1±0.4 | -1.8±0.4 | -1.4±0.5 |
